# Novel Clinical Updates in Uremia

**DOI:** 10.3390/jcm11133791

**Published:** 2022-06-30

**Authors:** Anna Clementi, Grazia Maria Virzì

**Affiliations:** 1 Department of Nephrology and Dialysis, Santa Marta and Santa Venera Hospital, 95024 Acireale, Italy; 2 IRRIV—International Renal Research Institute, 36100 Vicenza, Italy; 3 Department of Nephrology, Dialysis and Transplant, San Bortolo Hospital, 36100 Vicenza, Italy

The progressive loss of kidney function is responsible for the retention of different metabolites due to a decrease in their renal clearance. The majority of them affect cellular and organs’ function, resulting in the uremic syndrome. Uremia is characterized by fluid, electrolyte and metabolic anomalies. Patients presenting with uremic status typically complain of nausea, vomiting, fatigue, anorexia, weight loss, muscle cramps, pruritus, and changes in mental status.

The solutes involved in the pathogenesis of this clinical condition are called uremic toxins. Uremic toxins are reported to cause in vitro and in vivo harmful effects on several organs. Different organ-specific consequences may be induced by distinct molecular mechanisms or signal pathways. The best known retained solutes are creatinine and urea, which represent only two of the many solutes associated with the uremic syndrome, which is attributed to a much larger array of solutes. These compounds have been divided into different groups. Among them, there are advanced glycation end products (AGE), phenolic derivatives (including p-cresol), indole derivatives, hippurates, polyamines, peptides, homocysteine,3-carboxy-4-methyl-5-propyl-2-furanopropionic acid (CMPF), and trimethylamine-N-oxide(TMAO).

The European Uremic Toxin Work Group proposed a classification of the uremic toxins into three major groups based on their removal pattern by dialysis [1,2]: small water-soluble compounds, such as urea and creatinine; protein-bound compounds; the so-called middle molecules, which are mostly small peptides, such as microglobulins and complement. Many of the protein-bound solutes are actively transported from the plasma to the tubular lumen by organic anion or cation transporters in the proximal renal tubule. Among them, indoxyl sulfate, p-cresyl sulfate and kynurenine, all poorly dialyzable, accumulate as nephron function declines and might represent uremic toxins.

Evidence has demonstrated that indoxyl sulfate and probably other “uremic toxins” are not simply waste products, but they probably function as transcription factors [3]. Following entry through organic anion transporters, indoxyl sulfate binds to the aryl hydrocarbon receptor. This is followed by the release of heat-shock proteins and the passive transport of the complex of indoxyl sulfate/aryl hydrocarbon receptor across the nuclear membrane to bind with the aryl receptor nuclear translocator. This complex binds to specific xenobiotic response elements, on chromosomes, to activate gene expression. Overall, uremic toxins can turn on signaling pathways upon entering the cell and modulate the cellular response, contributing to the pathological process of chronic kidney disease (CKD).

Uremic toxicity is also cardiovascular toxicity. Inflammation, oxidative stress, and decreased nitric oxide availability are the main factors affecting cellular processes in CKD. Loss of endothelial function, proliferation of vascular smooth muscle cells and calcification, leukocyte activation, CKD-mineral bone disorder, insulin resistance, and increased thrombogenicity are all induced by uremic toxins. All these factors are involved in the pathogenesis of cardiovascular damage in CKD. Several studies in different in vitro cell models (i.e., HUVECs, RBCs, HASMCs, THP-1) have found that uremic toxins, such as indoles, p-cresylsulfate, phosphate, and urea, induce oxidative stress by expanding reactive oxygen species (ROS) production and triggering leukocyte free radical production, thus resulting in endothelial dysfunction and apoptosis [4].

Both cardiovascular disease and CKD, the so-called “cardiorenal or renocardiac syndrome”, are directly associated with increased cardiovascular morbidity and mortality [5]. In particular, CKD is responsible for changes in the structure and/or function of the heart referred to as “uremic cardiomyopathy”. Initially, this condition is associated with left ventricular hypertrophy and the characteristic fibrosis of the heart muscle, thickening of the wall of arterioles inside the heart muscle, and diastolic dysfunction, followed by systolic dysfunction, leading to overt heart failure at a later stage. Moreover, in patients with CKD, accelerated atherosclerosis is observed, and cardiac arrhythmias are frequently reported, in particular atrial fibrillation. Furthermore, uremic toxins also regulate signaling pathways and metabolism, potentially affecting gene expression in extra-renal and in extra-cardiac tissues. This entire system’s biological view of uremic toxins is leading to a new sight of uremia: uremic toxins are involved and cause aberrant organ crosstalk and affect extra-renal organs (e.g., gut–liver–kidney central nervous system) [6].

Importantly, drugs used in clinical treatments may affect the levels of uremic toxins, their tissue disposition, and even their elimination through the interaction of both with proteins such as albumin and cell membrane transporters. In this context, protein-bound uremic toxins (PBUTs) are highlighted for their high affinity for albumin, the most abundant serum protein with multiple binding sites and an ability to interact with drugs. Membrane transporters mediate the cellular influx and efflux of various uremic toxins, which may also compete with drugs as substrates, and both may alter transporter activity or expression [7].

The best-known options for overall toxin removal are dialysis and transplantation, which are part of the therapeutic options at the disposal of nephrologists. Perhaps one of the most convincing arguments in favor of a therapeutic outcome benefit is observed for hemodialysis, hemodiafiltration (a combination between dialysis and ultrafiltration) with large-pore (high-flux) hemodialysis membranes, or peritoneal dialysis.
